# Dr. Lyman L. Beck

**Published:** 1916-02

**Authors:** 


**Dr. Lyman L. Beck** (name given as Deck in State Directory), Michigan 1878, died at his home in Salamanca Jan. 3.

